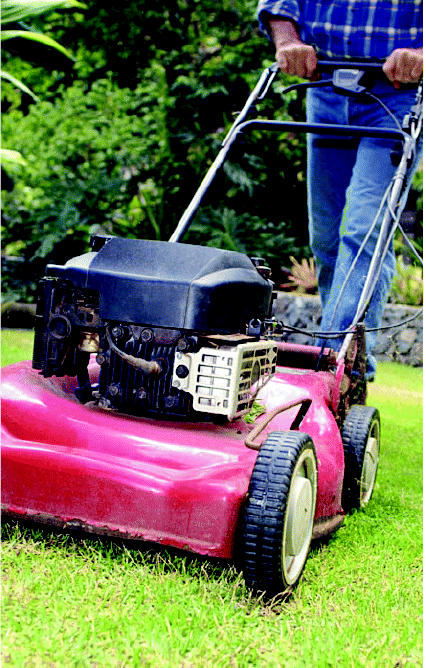# The Beat

**Published:** 2005-07

**Authors:** Erin E. Dooley

## “Cabin Fever” Fears Unfounded

Ever wonder how many infectious organisms are riding along with you in the cabin of a commercial airliner? According to a literature review in the 12 March 2005 *Lancet*, although airliner cabins are a suitable milieu for the spread of disease, the environmental control systems used by commercial aircraft appear to restrict the movement of airborne microbes. The review found that proper ventilation within cabins with one air exchange reduces concentrations of airborne organisms by 63%. Data models from a study of tuberculosis transmission aboard aircraft showed that doubling cabin ventilation rates reduced the infection risk by half. The paper’s authors point out that the fear of catching an infectious disease from a fellow passenger is greater than the actual risk.

## Betting on Biomass

The U.S. Department of Energy has unveiled a $2.85 million Biomass Surface Characterization Laboratory within the National Renewable Energy Laboratory in Golden, Colorado. Dedicated in March 2005, the new lab is designed to give scientists the means to make significant breakthroughs in the development of biomass as a viable energy source. The facility features the most advanced research tools to study biomass-to-energy processes at the atomic and molecular levels. One area the laboratory will explore is the creation of new technologies for biorefineries, which will produce bio-based transportation fuels and various other products the way petroleum refineries do today.

## Turning Up the Heat Watch

According to National Weather Service (NWS) data, excessive heat is the leading weather-related cause of death, with at least 1,500 excess deaths from heat-related causes during the average U.S. summer. The NWS has been testing its Heat/Health Watch Warning System to provide the public with five days’ advance notice of excessive heat events. Now the NWS has announced that the system, which has become a model for others worldwide, will be expanded to include every U.S. city with a population exceeding 500,000. In Philadelphia, the first city to implement the system, 117 lives were saved over three years.

## Japan Revs Up Idling Law

Since 1997 Japan has required that drivers of commercial vehicles turn off their engines when they were going to be stopped for more than just a few moments—for example, at curbs and stoplights. Now, thanks to studies proving the measures’ effectiveness in reducing carbon dioxide emissions and saving fuel, Japanese leaders plan to spread the movement to include private car owners. The Japanese Environment Agency calculates that if all 68.6 million cars owned in Japan idled for one less minute per day, more than 225,000 fewer tons of carbon dioxide would be emitted and 350 million liters of fuel would be saved.

## Africa Forms Waste Institute

Despite international conventions to control the importation and transboundary movement of hazardous waste, African nations still struggle with huge problems of pesticide dumps and illicit trade in hazardous waste. Now 10 nations have signed an agreement under the auspices of the Basel Convention to establish an African institute to deal with waste issues. The institute will be hosted by South Africa, and will be legally established once five states ratify the agreement to create it. The institute will develop training programs for the environmentally sound management of hazardous and other wastes, as well as facilitate the transfer of technologies in this area. Work is under way to set up similar institutes for other areas of Africa.

## The Lawn and Short of Mower Pollution

Not all lawn mowers are created equal, nor is all lawn mower pollution. From traditional gas-powered mowers to electric models, the amount of pollution produced varies significantly. A life cycle analysis done by University of Florida engineers confirms that gas-powered mowers produce more smog-forming pollution than their electric-powered counterparts. However, significantly more carcinogens and other toxicants may come from the manufacture and disposal of the batteries used in cordless electric mowers. Corded electric mowers, whose lifetime pollution consists of power plant emissions, were deemed least polluting of those tested.

## Figures and Tables

**Figure:**
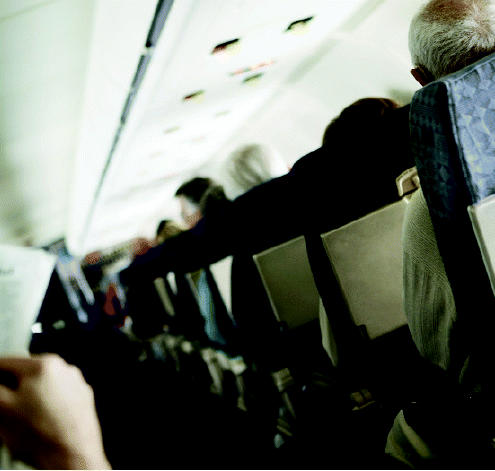


**Figure:**
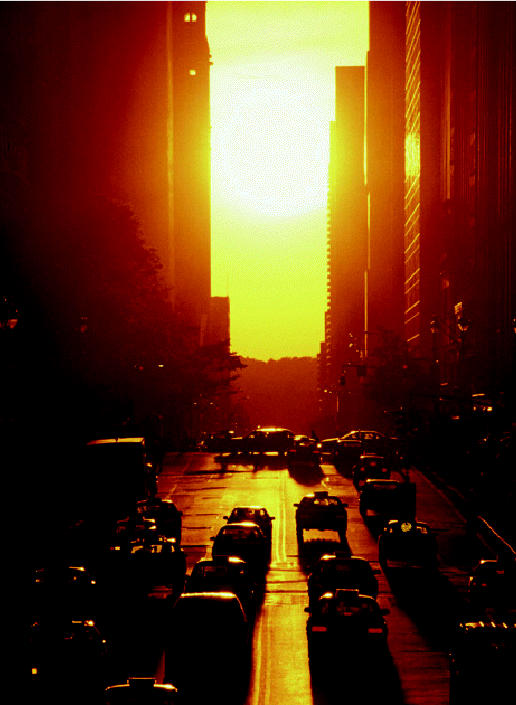


**Figure:**
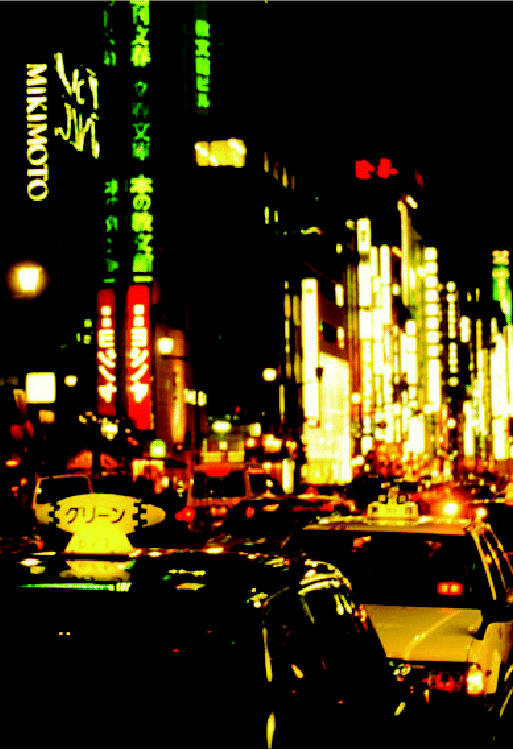


**Figure:**